# Molecular epidemiology of canine norovirus in dogs from Portugal, 2007–2011

**DOI:** 10.1186/1746-6148-8-107

**Published:** 2012-07-09

**Authors:** João Rodrigo Mesquita, Maria São José Nascimento

**Affiliations:** 1 Departamento de Ciências Biológicas, Laboratório de Microbiologia, Universidade do Porto, Portugal, Rua Jorge Viterbo Ferreira, 228, Porto, 4050-313, Portugal; 2 Secção de Ciências Veterinárias, Instituto Politécnico de Viseu, Quinta da Alagoa - Estrada de Nelas, Ranhados, Viseu, 3500-606, Portugal

**Keywords:** Canine norovirus, Dog, Fecal shedding, Winter, Seasonality

## Abstract

**Background:**

Canine noroviruses (NoVs) have been recently described in south European countries and associated with outbreaks of diarrhea in kennels. Unlike human NoV which are known as an important cause of acute gastroenteritis, little is known about the role of canine NoV as pathogens in dogs as well as its epidemiological features.

**Results:**

From 2007–2011, 256 stool samples were collected from dogs across Portugal and tested by RT-PCR for canine NoV. Viral fecal shedding was found to be 23% (60/256). All sequences contained the GLPSG amino acid motif characteristic of the RNA-dependent RNA-polymerase gene of NoVs and had a high nucleotide identity (range 98%–100%) to the canine NoV first described in Portugal. The highest shedding rate was detected during the winter months.

**Conclusions:**

This study shows that canine NoV infection is endemic in the dog population of Portugal. Peak shedding was detected in the winter months, a well-known epidemiologic feature of human NoV infections.

## Background

Noroviruses (NoV) are non-enveloped, single-stranded RNA viruses belonging to the genus *Norovirus* of the family *Caliciviridae*[1]. Until recently, NoV could be genetically classified into 5 genogroups (GI, GII, GIII, GIV and GV) with human NoV belonging to GI, GII, and GIV [1]. Human NoVs are the most frequent cause of outbreaks of acute gastroenteritis and the most common cause of sporadic enteric illness [2,3]. Infections occur year round but in temperate climates outbreaks show a seasonal peak activity during the winter [4]. Recently, NoVs were reported in dogs with acute gastroenteritis [5,6]. Canine NoV closely related to genogroup IV viruses was described in a diarrheic pup in Italy [5] and also caused an outbreak of diarrhea in a kennel in Greece [7]. A genetically different canine NoV was detected in dogs with diarrhea in Portugal [6]. Last year, we described an outbreak of gastroenteritis associated with canine NoV among kenneled dogs in Portugal, highlighting for the first time some epidemiological similarities between canine and human NoV outbreaks [8]. Despite the focus on the potential role as a pathogen in dogs, epidemiological features of canine NoV are not well described. Additionally, although a world-wide distribution of canine NoV has been suggested [9], the extent of geographical dissemination of canine NoV in different dog populations is unknown. In the present work, we tested fecal specimens from dogs collected from 2007 and 2011 in Portugal. Our data suggest seasonal variation of canine NoV infections similar to human NoV infections.

## Methods

### Samples

Between 2007 and 2011, a total of 256 fecal samples were collected from dogs (regardless of their health status) housed in a variety of facilities such as municipal dog pounds, non-profit dog rescue shelters, animal shops, veterinary hospitals and veterinary clinics across Portugal.

### RNA extraction and RT-PCR

Stools were diluted (10%) in phosphate-buffered saline, pH 7.2, and solids were removed by centrifugation at 8000 g for 5 min. Viral nucleic acid was extracted from 140 μl of each clarified stool suspension by the QIAamp viral RNA mini kit (Qiagen, Hilden, Germany), according to the manufacturers’ instructions. Viral nucleic acid was tested for the presence of canine NoV by RT-PCR (Qiagen One-Step RT-PCR Kit) using the primers JV102 (5′-TGG GAT TCA ACA CAG CAGAG-3′) and JV103 (5′-TGC GCA ATA GAG TTG ACCTG-3′) targeting a small region of the RNA-dependent RNA-polymerase gene [6]. Briefly, 5 μl of viral nucleic acid was added to an RT-PCR mix (Qiagen One-Step RT-PCR Kit) containing RNase-free water, Qiagen RT-PCR buffer (5x), dNTP mix (10 mM), Enzyme mix (RT and Taq), 100 mM forward and reverse primer and RNase inhibitor (40 U). Cycling conditions consisted of reverse transcription for 30 min at 42°C, activation of Taq polymerase for 15 min at 95°C, 40 cycles of 1 min at 94°C, 1 min at 37°C, and 1 min at 72°C, followed by a final extension for 10 min at 72°C.

### Sequencing and phylogenetic analysis

RT-PCR products were separated by electrophoresis in a 1.5% agarose gel and visualized under UV after ethidium bromide staining. Appropriately sized bands (215 bp) were excised from the gel and purified with the QIAquick gel extraction kit (QIAGEN) and sequenced in both directions using the BigDye Terminator v1.1 Cycle Sequencing kit (PE Applied Biosystems, Foster City, CA, USA). Sequence editing and multiple alignments were performed with the BioEdit software package, version 2.1. Phylogenetic analysis was drawn by using TreeCon software with bootstrap analysis (n = 1,000), and the tree topology was inferred by using neighbor-joining.

## Results

**Figure 1:**
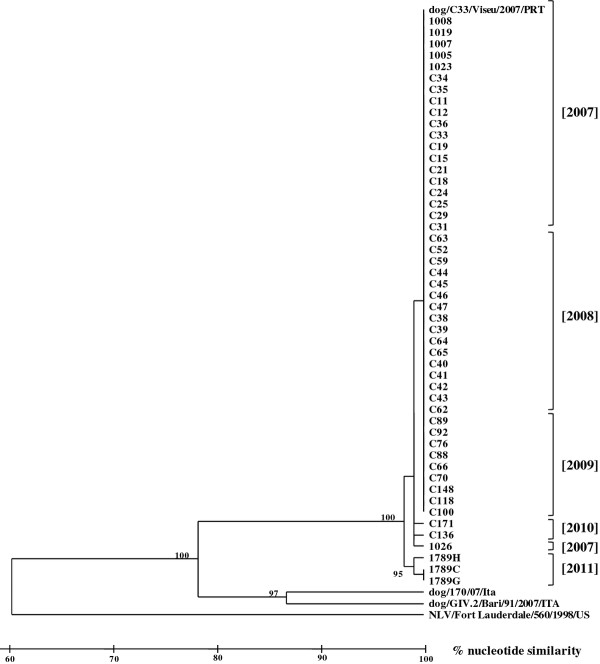
**Phylogenetic tree of a 215-nt region of the RNA-dependent polymerase gene of one human genogroup IV strain NLV/Fort Lauderdale/560/1998/US (accession no: AF414426), the two canine noroviruses reported from Italy, dog/170/07/Ita (accession no: EU224456) and dog/GIV.2/Bari/91/2007/ITA (accession no: FJ875027), and the canine norovirus reported from Portugal dog/C33/Viseu/2007/PRT (accession no: GQ443611).** Sample collection years are in brackets.

Sixty (23%) of the 256 samples tested positive for canine NoV. Sequences from the 60 samples contained the GLPSG amino acid motif characteristic of NoV viral RNA-dependent RNA-polymerase. Sequence analysis showed a high nucleotide identity (range 98%–100%) to the first canine NoV detected in Portugal, Viseu strain [6] (Figure 1).

**Figure 2:**
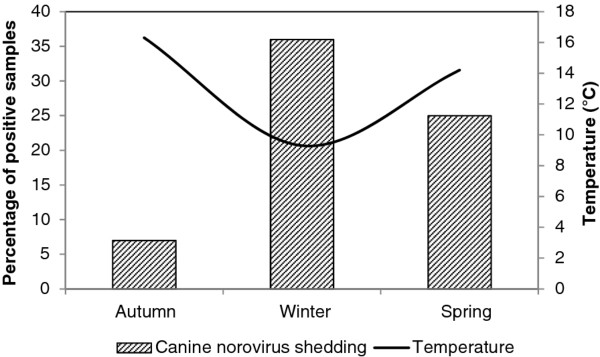
**Seasonal variation of canine norovirus fecal shedding in dogs of Portugal from 2007 to 2011 and mean temperature in Portugal from 2000–2009[**[10]**].**

From a total of 91 dogs tested in winter months, 33 (36%) were found to be shedding canine NoV, whereas only 25% (21/84) and 7% (6/81) were shedding canine NoV in spring and autumn months, respectively (Figure 2). A chi-square test for homogeneity of proportions (SPSS 13.0, SPSS Inc., Chicago, IL, USA) showed that the percentage of positive samples in autumn, winter and spring months were significantly different (p <0.001).

## Discussion

During a 4-year period 23% of stools collected from dogs across Portugal tested positive for canine NoV. Compared to the prototype canine NoV (Viseu strain), all strains had a high identity at nucleotide (range 98%–100%) and amino acid (100%) levels. At the nucleotide level several strains from 2007, 2008 and 2009 had identical sequences to the Viseu strain. Interestingly, strains detected in 2010 had an identical sequence as strain 1026 collected in 2007 tempting us to speculate that strain 1026 might have emerged as a new genetic variant and prevailed through 2010 in the dog population. However, because only a small fragment of the genome was sequenced these conclusions should be interpreted with caution and additional sequence information from the capsid regions of the genome is required to confirm this hypothesis.

The mechanism how new NoV variants emerge is currently unknown. For human NoVs, the high genetic mutation rate [11], as well as viral antigen and host receptor interactions [12] have been suggested as potential contributors. Similar events could have taken place for canine NoV in the dog population.

Most canine NoV positive samples had been collected during winter months. Although no samples were collected during the summer months, our data show a peak of canine NoV activity in the colder months, an epidemiological feature typical of human NoV infections [13]. Additional studies with at least monthly sampling for several years are necessary for a more comprehensive analysis. The reason for human NoV seasonality remains unknown, but is believed to be a combination of complex multifactorial aspects that include environmental, host and virologic factors [14]. Changes in environmental conditions, such as humidity and temperature, are known to be associated with seasonality of viral diseases allowing for a winter predisposition [15]. The seasonality of rotavirus has been suggested to be due in part to the low relative humidity indoors during the cold periods, encouraging not only the persistence of infectious viruses on surfaces but also aerosolization of virus-laden dust particles [16]. Concerning NoV, it is known that cool and dry conditions are favorable for survival of infectious virus [17]. An increase in the number of canine NoV positive samples in the colder months might be associated with the overcrowding of kennels, a typical event in rainy and colder winter months where animals are more often kept indoors. Enteric virus transmission in dogs has been mentioned to be facilitated in large breeding colonies where hygiene is difficult to maintain and fecal contamination of the environment is at its maximum [18]. Moreover, seasonal variations in the host susceptibility to infections have also been suggested, possibly associated with changes in the animals’ physiological status in winter [18]. Hence, more thorough kennel hygiene and disinfection procedures should be taken in the colder months when overcrowding is likely to occur, in order to decrease the chances for transmission of canine NoV.

## Conclusions

This study shows that canine NoV infection is endemic in the dog population of Portugal. Peak shedding was detected in the winter months, a well-known epidemiologic feature of human NoV infections. This interesting finding is preliminary and requires to be further explored. Overall, the present work constitutes an important contribution to the knowledge of the epidemiology of canine NoV infection, still at its infancy.

## Competing interests

The authors declare that they have no competing interests.

## Authors’ contributions

JRM and MSJN conceived and designed the study, drafted and revised the manuscript. JRM performed the laboratory assays. Both authors approved the final manuscript.
